# Catalytic Low-Temperature Dehydration of Fructose to 5-Hydroxymethylfurfural Using Acidic Deep Eutectic Solvents and Polyoxometalate Catalysts

**DOI:** 10.3389/fchem.2019.00661

**Published:** 2019-10-09

**Authors:** Sam Körner, Jakob Albert, Christoph Held

**Affiliations:** ^1^Laboratory of Thermodynamics, TU Dortmund University, Dortmund, Germany; ^2^Lehrstuhl für Chemische Reaktionstechnik, Friedrich-Alexander Universität Erlangen-Nürnberg, Erlangen, Germany

**Keywords:** biomass dehydration, carboxylic acid, alkyl ammonium, HMF, reaction equilibrium

## Abstract

HMF synthesis typically requires high temperature and is carried out in aqueous solutions. In this work, the low-temperature dehydration of fructose to HMF in different deep eutectic solvents (DES) was investigated. We found a very active and selective reaction system consisting of the DES tetraethyl ammonium chloride as hydrogen bond acceptor (HBA) and levulinic acid as hydrogen bond donor (HBD) in a molar ratio of 1:2 leading to a maximum HMF yield of 68% after 120 h at 323 K. The DES still contained a low amount of water at the initial reaction, and water was also produced during the reaction. Considering the DES properties, neither the molar ratio in the DES nor the reaction temperature had a significant influence on the overall performance of the reaction system. However, the nature of the HBA as well as the acidity of the HBD play an important role for the maximum achievable HMF yield. This was validated by measured yields in a DES with different combinations of HBD (levulinic acid and lactic acid) and HBA (choline chloride and tetra-n-alkyl ammonium chlorides). Moreover, addition of vanadium containing catalysts, especially the polyoxometalate HPA-5 (H8PV5Mo7O40) leads to drastically increased reaction kinetics. Using HPA-5 and the DES tetraethyl ammonium chloride—levulinic acid we could reach a maximum HMF yield of 57% after only 5 h reaction time without decreasing the very high product selectivity.

## Introduction

The chemical industry of the future will have to move from decades of fossil energy sources, such as coal and oil, to more sustainable organic resources. Biomass has the greatest substitution potential for fossil raw materials among renewable energy sources. Due to the decreasing availability of these resources and important intermediates, interest in cost-effective biomass conversion processes into valuable platform chemicals is growing. In particular, the energy-efficient and economical dehydration of monosaccharides is the focus of attention (Chheda et al., 2007; Liu et al., 2012).

5-Hydroxymethylfurfural (HMF) is a biomass-based key component in the biological pathways of a variety of high-value platform chemicals. Due to its high functionality, HMF represents an ideal building block in the interface between carbohydrate and petrochemicals. Both the hydroxymethyl group and the aldehyde group open up the possibility of various subsequent reactions to important platform chemicals (Bozell and Petersen, 2010). Within the derivatization of HMF, there is a particular interest in the resulting furan compounds. Especially furan dicarboxylic acid (FDCA) and levulinic acid (LA), which are included in the list of top 12 biomass-based chemicals with high-added-value published by the U.S. Department of Energy (Bozell and Petersen, 2010). FDCA is a promising alternative to petroleum-derived terephthalic acid. In addition to the normal market for polyethylene terephthalate (PET, 1.8 · 10^9^ tons per year), this substitution potential is gaining in importance with regard to increasing research activities in the direction of “bio-PET” (Werpy and Petersen, 2004; Iwata, 2015).

The acid-catalyzed synthesis of HMF occurs under triple elimination of water starting from fructose as a substrate (Tang et al., 2017). A suitable solvent as a reaction medium is essential for the thermodynamic optimum of an overall process. Due to the fact that often more than 80% of all chemicals in a process make up the solvents, the profitability also depends heavily on the choice of solvent. Therefore a wide variety of solvents, such as aqueous media (Vandam et al., 1986; Hansen et al., 2009), dimethyl sulfoxide and methyl isobutyl ketone (Yi et al., 2015) or biphasic reaction media (Chheda et al., 2007; Hu et al., 2008) and even in melts (Ilgen et al., 2009) have been extensively studied so far. However, either high reaction temperatures are required or the solvents are toxic and often also expensive. Further, from the thermodynamic point of view, water must be avoided as reaction solvent as the product of the reaction is also water; thus the latter as solvent strongly limits the yield of HMF. Ionic liquids (ILs) as more recent solvents are interesting in this respect (due in part to the physicochemical properties), but often also disadvantageous because of the high price or toxicity (Thi et al., 2010; Russ and Konig, 2012) or because of slow kinetics. Thus, alternative solvents are required in combination with new catalysts. ILs have already been successfully applied as reaction medium for fructose dehydration (D'Anna et al., 2014; Zhang et al., 2015).

In recent years, research on deep eutectic solvents (DESs) has been intensified. An eutectic mixture is characterized by the lowest melting temperature of all compositions of the same components (see Figure S3). At the eutectic point, the melting temperature is significantly lower than that of the pure substances. If mixtures have a very large melting point reduction compared to the pure substances, these are referred to as deep eutectic systems. Accordingly, DESs as the reaction medium allow for reaction temperatures near room temperature. DESs are composed of a hydrogen-bond acceptor (HBA) and a corresponding donor (HBD) (Abbott et al., 2004). Often the DESs consist of a quaternary ammonium salt as HBA and a wide range of HBDs. For the latter typically carboxylic acids, alcohols or amines are used (Russ and Konig, 2012). Moreover, DESs based on an acid can serve both as a solvent and as a catalyst. Additionally, depending on the combination of HBA-HBD, DESs might represent a green solvent that can replace organic solvents in engineering applications in the long term. Several DESs share many of the benefits and overcome some of the disadvantages of ILs. In addition to the simple and cost-effective production, especially a possible low toxicity as well as a possible good biodegradability and biocompatibility with enzymes are among the advantages. Furthermore, DESs are referred to as designer solvents because the properties can be tailored to the specific requirements, depending on the application (Smith et al., 2014). Product separation from the solvent is often possible by crystallization, which allows more energy-intensive processes to be avoided (Francisco et al., 2013). Therefore, in the interests of the eco-efficient health and safety industry, they offer enormous potential for improving ecotoxicity, waste management and process safety (Zhang et al., 2012). DESs have been also used as reaction medium for fructose dehydration to HMF (Istasse et al., 2018). The use of DESs in the synthesis of biomass-based platform chemicals is well in line with the twelve principles of Green Chemistry. DESs are becoming increasingly important due to their biodegradability, biocompatibility and very low toxicity. In addition, DESs allow for a significant lowering of the reaction temperature leading to lower energy consumption. This not only increases energy efficiency but also atomic efficiency, leading to a reduction of derivatives as the formation of by-products increases with increasing temperature (Zakrzewska et al., 2011). Also, the use of catalysts should be optimized as they are superior to stoichiometric reagents. The reader is further referred to review articles on the use of DESs in upstream and downstream processes (Smith et al., 2014; Sheldon, 2016; Vitale et al., 2017; Garcia-Alvarez et al., 2018).

Moreover, the work-up of the catalyst is frequently complicated and time-consuming (Yi et al., 2015). Typical catalysts for this reaction discussed in the literature are metal and organic acids as well as ion exchange resins (Carniti et al., 2011; Zakrzewska et al., 2011). Chromium compounds used as catalysts showed good results. However, their high toxicity associated with high loadings is an obstacle to commercial applications (Zakrzewska et al., 2011). In the future there will be an increased demand for catalysts for the dehydration of fructose to HMF on a large scale. The use of catalysts is closely linked to the reaction time and therefore an important point in terms of process optimization.

Another promising alternative class of catalysts for low-temperature homogeneous biomass transformation reactions is polyoxometalates (POMs). POMs are well-defined metal-oxyanions linked with oxygen bridges of early transition metals at their highest oxidation state (e.g., Mo^6+^, W^6+^ or V^5+^). They can also contain a multitude of hetero atoms to improve their chemical and thermal stability (Ammam, 2013). POMs reveal unique physical and chemical properties like tunable acid-base properties, great redox activity based on the fast and reversible multi-electron transfer, high thermal stability and excellent solubility and stability in water and other polar solvents (Albert et al., 2012; Wang and Yang, 2015). Mostly used in homogeneous catalyzed biomass transformation reactions are POMs from the so called Keggin-type [XM_12_O_40_]^n−^. They contain a template of various coordinating anions, e.g., oxo-anions, oxometalates, or halides, together with a framework metal which is typically an early, high-valent transition metal (Kozhevnikov, 1998). The catalytic activity is mostly introduced by substituting some of the framework metals (W, Mo) with easily reducible hetero-metals like vanadium that results in shifting their reactivity from acidic to redox-dominance (Albert et al., 2016). The generated compounds have the composition H_3+n_[PV_n_Mo_12−n_O_40_] and are called heteropolyacids, abbreviated as HPA-n depending on the content of vanadium atoms (n).

In this contribution, we investigate the low-temperature dehydration of fructose to HMF in different DESs. Therefore, we identified the best HBA:HBD combination leading to high HMF yields. Moreover, we studied the effect of using different vanadium-containing catalysts on the efficiency and the kinetics of the fructose dehydration reaction.

## Materials and Methods

### Materials

Chemicals that have been used in this work are listed in Table S8. The quaternary ammonium salts choline chloride, tetrabutyl, and tetraethyl ammonium chloride were dried for 24 h at 298 K in a vacuum oven before use. All other chemicals were used as obtained without further purification. Deionized water from the Millipore system (Merck KGaA, Darmstadt, Germany) was used.

Moreover, three vanadium-containing acid catalysts were used in this study. The vanadium precursor NH_4_VO_3_ (CAS-Nr. 8037803-55-6) was purchased from Acros Organics with a purity of 99.5%. VOSO_4_ (CAS-Nr. 123334-20-3) was obtained from Alfa Aesar with a purity of 99.9%. Moreover, we used the heteropolyacid HPA-5 (H_8_PV_5_Mo_7_O_40_) that was synthesized in the Erlangen labs according to the literature (Odyakov and Zhizhina, 2008; Albert et al., 2014). The characterization of the heteropolyacid has been carried out using a Perkin Elmer Plasma 400 ICP-OES device resulting in a P/V/Mo ratio of 1/4.80/6.93 as well as FTIR spectroscopy (Jasco FT/IR-4100 spectrometer equipped with a PIKE GladiATR-accessory) showing the characteristic vibrations at wavenumbers of 1,045 (Mo = O), 948 (P-O), 864 (Mo-Ob-Mo) as well as 725 (Mo-Oc-Mo) cm^−1^.

All powdered catalysts were finally dried at 10^−3^ mbar by keeping a constant low temperature of max. Two hundred and seventy seven Kelvin in order to adjust a fixed content of hydration water. Thermogravimetric analyses (TGA) were conducted using a SETSYS-1750 CS Evolution from SETARAM Instruments, resulting in water contents of 2 moles for NH_4_VO_3_, 4 moles for VOSO_4_, and 12 moles for the HPA-5 catalyst, respectively.

### Synthesis of Deep Eutectic Solvents

The various DESs were prepared in a molar ratio of 1:2. For this purpose, two moles of the carboxylic acid per mole of the quaternary ammonium compound were weighed into one 10 ml Falcon tube. The DESs were prepared gravimetrically using a Sartorius laboratory balance Cubis (Sartorius, Goettingen, Germany) with an accuracy of ±10–4 g. The tubes were placed in the ThermoMixer^®^ (Eppendorf, Hamburg, Germany) at 373 K and 1,000 rpm for a few minutes until a homogeneous liquid was obtained.

### Solid-Liquid Equilibria of the Deep Eutectic Systems

In order to allow liquid reactions at 323 K, the solvent (in this work: DESs) have to be a homogenous liquid. The phase diagram was measured in this work in order to ensure homogenous liquid phase conditions at the desired reaction temperature of 323 K at 1 bar. For this purpose, solid-liquid equilibria were investigated by means of measurements and validation by thermodynamic modeling. In the experimental work, HBD (levulinic acid) and HBA (choline chloride) were prepared in different molar ratios by weighing HBD and HBA as desired into 10 ml Falcon tubes gravimetrically using a Sartorius laboratory balance Cubis (Sartorius, Goettingen, Germany) with an accuracy of ±10^−4^ g. These mixtures were placed in a Thermomixer C at 373 K and shaken at 300 rpm. All considered mixtures were homogenously liquid at 373 K. Afterwards, temperature of the Thermomixer block was reduced by 5 K, and shaken again for another 24 h. In case heterogeneity was observed during this time period the temperature was assumed to be the solubility temperature of the samples at 1 bar and known composition. In any other case the temperature was reduced by another 5 K and the procedure was repeated accordingly. This method is not very accurate, and the uncertainty in the measured solubility temperature is 5 K. Thus, the measured phase diagram was predicted a priori with the thermodynamic model PC-SAFT. All available PC-SAFT parameters of the pure components were taken from literature [choline chloride (Zubeir et al., 2016), lactic acid (Zubeir et al., 2016), and levulinic acid (Altuntepe et al., 2017)]. Binary parameters were not used. Details of the model and the solid-liquid equilibrium condition can be found in (Crespo et al., 2018).

### Reaction Equilibrium Experiments and Analysis

In each case 1 g of the different DESs was transferred by syringe into a 10 ml Falcon tube and 2.5 wt.% fructose was added. The amount of fructose was adapted to the lowest experimentally determined fructose solubility in the different DESs. Every initial reaction mixture was prepared twice, so that each experimental reaction equilibrium position was obtained by dual approach. The reaction mixtures were prepared gravimetrically using a Sartorius laboratory balance Cubis (Sartorius, Goettingen, Germany) with an accuracy of ±10–4 g. The tubes were shaken in the ThermoMixer^®^ (Eppendorf, Hamburg, Germany) at 323 K and 1,000 rpm. Thereafter, samples were taken at solvent-specific intervals over an extended period of time and analyzed by HPLC. The length of the period depended on the time required to reach the reaction equilibrium.

Equilibrium concentrations of the reacting agents were analyzed using two different analytical methods. For this purpose, an Agilent series 1,200 HPLC (Agilent, Böblingen, Germany) equipped with a refractive index detector (RID) was used to detect the key components HMF and fructose. Sampling from the vial and subsequent injection of 5 μl of the sample in the chromatography column was fully automatic by a built-in syringe. For the separation a Nucleogel^®^ Sugar 810 H column (300 × 7.8 mm, Macherey Nagel, Düren, Germany) thermostated at 308.15 K and at a flow rate of 0.6 ml/min was used. The stationary phase was a polymer phase suitable for the separation of a wide range of organic acids, alcohols and sugars. The mobile phase was a mixture of sulfuric acid (5 mmol/l) and water that was used isocratically (sulfuric acid:water = 1:20). The calibration for the key components was carried out for at least three different mass fractions in the range from 0.00 to 13.05 wt.%. Each sample and calibration solution was prepared twice and analyzed at least three times. The experimentally determined retention times are listed in Table S1. If the components were solid at room temperature (298 K), they were dissolved in deionized water. Two chromatograms are given as an example in Figures S1, S2.

The evaluation of the water peak was not possible at the given conditions due to overlays through similar retention times of other substances. Therefore, the equilibrium concentration of water was determined by means of the Karl Fisher titration (KFT) with a Metrohm 915 KF Ti Touch (Metrohm, Herisau, Switzerland). Each sample was measured at least three times and the results were averaged. The results are given in Table S9.

If the fructose peak was not evaluable due to overlay, the concentration in the reaction equilibrium was estimated from the molar balance using the initial molalities, the measured molality of HMF and water at equilibrium as well as the stoichiometry of the reaction.

Further, the chemical stability the carboxylic acids at reaction conditions (323 K at 1 bar as well as 343 K at 1 bar) was verified by HPLC-measured peak areas of the carboxylic acids (levulinic acid and lactic acid) after exposure to reaction conditions for up to 300 h (this was the maximally applied reaction time).

### Reaction Experiments Using Additional Vanadium-Based Catalyst

Several days may pass before the reaction equilibrium is reached in DESs, which ideally serve both as a solvent and as a catalyst. Because of the slow kinetics, the use of additional catalysts in the most promising DES has been studied. For this purpose, three selected catalysts were used. The powdered catalysts used included NH_4_VO_3_, VOSO_4_, and HPA-5. The catalysts were added both in powder form and dissolved in water to the DES. Regardless of whether the catalyst was present as a powder or in solution, the weight of pure catalyst in all batches was 4.0 wt.%. This corresponded to the lowest solubility of any of the undissolved powdered catalysts in the DES used. The time intervals of sampling and analysis were adjusted to the expected higher reaction rate. Otherwise, the experimental procedure was as described in the previous section.

## Results and Discussion

**Scheme 1:**
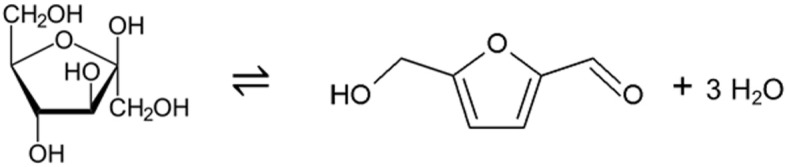
Reaction considered in this work: Dehydration of D-(-)-fructose to 5-hydroxymethylfurfural (HMF) and three water molecules.

In this work, the reaction of fructose to HMF was studied (Scheme 1) using the chemicals as listed in Table S1.

### Choice of the Reaction Temperature

In order to define a meaningful temperature for fructose dehydration in DESs, the influence of the reaction temperature on the HMF yield was measured in the DES ChCl:LA. Two independent DES samples were synthesized, both in a molar ratio of 1:2. The reaction mixtures were stirred at 323 K as well as at 343 K. These temperatures were chosen as we wanted to have a temperature near to ambient conditions in order to minimize the energy required and to minimize side reactions, as it was found that temperatures higher than 343 K cause losses in selectivity (Assanosi et al., 2014). Table 1 shows the yields of HMF at these different reaction temperatures.

**Table 1:** 5-Hydroxymethylfurfural (HMF) yields in choline chloride:levulinic acid (ChCl:LA) in a molar ratio of 1:2; reaction temperature: 323 or 343 K; fructose starting concentration: 2.5 wt.%; pressure: 1 bar.

**Time [h]**	**ChCl:LA (323 K)**		**ChCl:LA (343 K)**	
	**Y_**HMF**_ [%]**	**SD [%]**	**Y_**HMF**_ [%]**	**SD [%]**
24	18	–	21	0.3
120	48	3.3	45	4.3

*Standard uncertainty: u(T) = 0.5 K; u(Y_HMF_) = 1.7%*.

At a reaction temperature of 323 K, an HMF yield of 18% after 24 h and of 48% after 120 h was observed. At 343 K, the yield of HMF increased from 21% after 24 h to 45% after 120 h. Thus, on the basis of these measurement results, increasing the reaction temperature to 343 K is not beneficial for the HMF yield. It should be noted that this depends also on the nature of the solvent. For the reaction carried out in ILs, a more significant temperature effect on the kinetics of the reaction was observed in the literature (Okano et al., 2013). For the reaction in the DESs under investigation in this work, increasing temperature to 343 K did not lead to the desired improvement in reaction kinetics considering the rather small temperature range (323 K to 343 K). One reason might be a phase separation that was observed after the reaction at 343 K and cooling to room temperature, and the brownish color of the sample let assume polymerization of reactants and intermediates as well as production of humins. This might also explain that the yield is more or less the same at 343 K and at 323 K after 120 h of reaction time, in contrast to expectations based on Arrhenius. Therefore, 323 K was selected as the preferred reaction temperature for the following investigations. This decision was especially based on the fact that high selectivity was reached according to the chromatograms in Figures S1, S2.

### Fructose Dehydration to HMF in Various Reaction Media

As to be seen from Scheme 1, water as a reaction medium is undesired as it will shift the reaction equilibrium to the left hand side and thus will limit the HMF yield. Consequently, the use of DES as green reaction solvents was studied in this work. Non-aqueous reaction media such as the DESs do not have this shortcoming, and potentially should allow a much higher HMF yield compared to aqueous reaction medium. It should be noted that in the present study fructose was used as reactant at rather low concentration, which will not lead to formation of a DES based on fructose. To further limit the range of DESs that might be suitable as reaction solvent, several criteria were defined: First, the DES should be homogenously liquid at 323 K. Further, systems of low viscosity were preferred to reduce energy required for mixing. In order to avoid addition of acid which might be required as dehydration of fructose to HMF is known to be acid-catalyzed, carboxylic acids were chose as HBDs. Lactic acid can be synthesized by the fermentation of starch and levulinic acid by the hydrolysis of cellulose, thus both of them were tested as biogenic DES constituents. Although the use of salts might be critically seen for a greener chemistry in the future, the reaction of fructose to HMF seems to be favored under the presence of chloride. Thus, choline chloride (ChCl) was chosen as HBA DES constituent due to its biodegradability, biocompatibility and very low toxicity. Choline chloride is technically used in food supplements and feeds. As the DESs based on choline chloride and carboxylic acids are liquid and of extremely low volatility, low reaction temperatures are possible that opens the door for energy-efficient processes. These DESs further have a comparably low viscosity (Zhao et al., 2015; Li et al., 2016).

**Figure 1:**
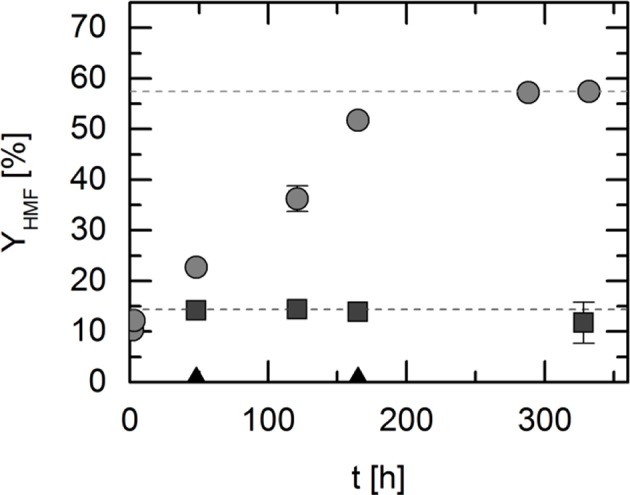
HMF yield over time in choline chloride (ChCl) dissolved in water (triangles), in pure levulinic acid (LA; squares) and in the corresponding deep eutectic system (DES ChCl:LA 1:2; circles); fructose starting concentration: 2.5 wt.%; reaction temperature 323 K; pressure 1 bar. Data listed in Table S2.

The studied DESs were chosen at a composition which allows liquefied system conditions at 323 K. The solid-liquid equilibria of the systems under consideration are presented in Figure S3. All systems were found to be liquid at the reaction temperature 323 K. The DESs are mixtures of two components. The hypothesis that should be verified in this work is that the combination of the two DES constituents to form a close-to-eutectic mixture yields solvent properties for the reaction that is very different from the solvent composed of only one of the DES constituents. Thus, the DESs as well as the individual constituents of the DESs were used as reaction solvents. The DESs as well as the individual constituents of DES have been studied with focus on HMF yield, and the following reaction solvents were analyzed: ChCl was dissolved in water (ChCl is a solid up to 575 K), pure levulinic acid (LA), and finally the DES consisting of these components (ChCl:LA) in a molar ratio of 1:2. These experiments indicated that the individual DES components are poorly suitable as reaction medium, whereas the DES consisting of these constituents are highly promising. Indeed, this supports the hypothesis that DESs act reaction solvent in a different way than its constituents. The most probable reason behind this is the interactions between reacting agents and the DES, which are different compared to the interactions between reacting agents and a single DES constituent. The yield of HMF over time in these different solvents is shown in Figure 1.

Kinetics and yield of chemical and biochemical reactions strongly depend on the reaction solvent (Lemberg and Sadowski, 2017; Voges et al., 2017; Wangler et al., 2019). Figure 1 shows that this was also observed for the reaction under consideration in the present study. After 48 h, an HMF yield of 14% was achieved in pure LA. This yield remained unchanged even after 328 h and thus corresponds to the maximum yield, which is the value at equilibrium assuming no side reactions or consecutive reactions. However, there was no reaction in the aqueous ChCl solution observable. This shows that neither aqueous ChCl, nor pure LA promote the reaction sufficiently. In comparison, using the DES ChCl:LA in a molar ratio of 1:2, which is already liquid at about 323 K (see Figure S3), HMF yields of 23% after 48 h and 57% after 288 h were achieved. The latter corresponds to the yield of HMF in equilibrium. The positive effect on the HMF yield by using the DES compared to the pure substances confirms the hypothesis on the synergetic effect that is the driving force for the reaction. Thus, HMF yields could be achieved, which exceeded those in the pure substances by up to 57%. However, it has to be mentioned that under the mild reaction conditions of 323 K and ambient pressure, the dehydration reaction in the DES is still very slow, as no additional catalyst was used to improve the kinetics. Moreover, HPLC analysis confirmed the very high selectivity of the reaction, as no other byproducts could be detected within a retention time of 1 h. Two chromatograms are given as an example in Figures S1, S2.

### Influence of Molar Ratio in the DES

Obviously, a mixture of ChCl and carboxylic acid is superior over using the individual DES constituents with respect to HMF yield. In Figure 1, this was demonstrated using the ratio of 1:2 (ChCl:LA). To analyze the influence of the molar ratio of the DESs on the HMF yield, two DES mixtures were synthesized, one in a molar ratio of 1:2 (ChCl:LA) and the other in the ratio 1:3 (ChCl:LA), respectively. Table 2 shows the experimentally observed yields of HMF at these considered molar ratios of the DES constituents.

**Table 2:** 5-Hydroxymethylfurfural (HMF) yields in choline chloride:levulinic acid (ChCl:LA) in a molar ratio of 1:2 and 1:3; reaction temperature: 343 K; fructose starting concentration: 2.5 wt.%; pressure: 1 bar.

**Time [h]**	**ChCl:LA (mr 1:2)**		**ChCl:LA (mr 1:3)**	
	**Y_**HMF**_ [%]**	**SD [%]**	**Y_**HMF**_ [%]**	**SD [%]**
24	21	0.3	24	2.9
120	45	4.3	50	2.1

*Standard uncertainty: u(T) = 0.5 K; u(Y_HMF_) = 1.7%*.

In the DES ChCl:LA with a molar ratio of 1:2, the yield of HMF increased from 21% after 24 h to 45% after 120 h. The HMF yield in the DES ChCl:LA with a molar ratio of 1:3 was 24% after 24 h, and 50% after 120 h, respectively. From this it is evident that there is no significant correlation between the molar ratio within the DESs and the HMF yield. Also the viscosities of the pure DESs ChCl:LA 1:3 [viscosity about 134 mPas (Li et al., 2016)] and ChCl:LA 1:2 [viscosity about 119 mPas (Zhao et al., 2015)] are very similar and mass-transport limitations are not expected to have a major influence. As the differences in viscosity and HMF yield as well as in kinetics are not very pronounced, a molar ratio of 1:2 was chosen as DES solvent for the following experiments. This decision was mainly based on the fact that other DESs with a ratio of 1:2 were analyzed as well (see section Influence of the Chemical Nature of the HBA in DESs With Levulinic Acid) and the results will be compared on the basis of a constant molar ratio of the different DESs under consideration.

### Influence of Acidity and pH

**Figure 2:**
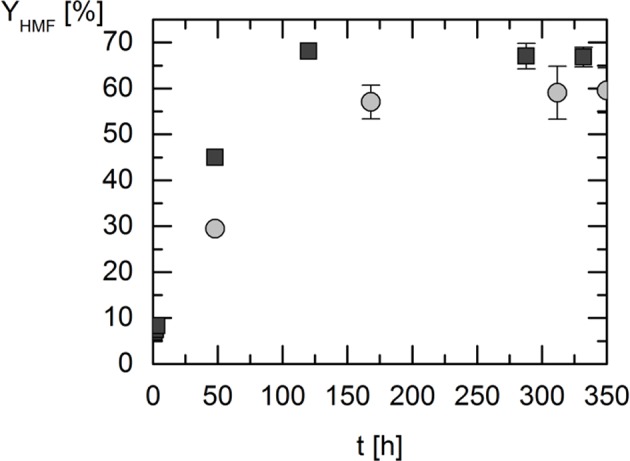
5-Hydroxymethylfurfural (HMF) yield over time in the different deep eutectic systems based on tetraethyl ammonium chloride (TEAC); reaction temperature: 323 K; w_FRU,0_ = 2.5 wt.%; pressure: 1 bar. Circles: TEAC:lactic acid 1:2; squares: TEAC:levulinic acid 1:2. Data listed in Table S3.

Dehydration of fructose to HMF is an acid-catalyzed reaction. Thus, by using a carboxylic acid as HBD, the DESs operate in functions of reaction medium as well as of the catalyst by assuring a certain required (low) pH. Previous works have shown that pH alone decides whether the reaction takes place or not. It was found that HMF is not formed at pH > 3.9 (Kuster and van der Baan, 1977), which fits to the observation that LA is a too weak acid for acting as a catalyst. This was found by Carniti et al. (2011), and also within this work. pH of LA was measured to be about pH = 4. ChCl-water mixtures are by far not acidic enough to catalyze the reaction, as at 323 K the pH values of these mixtures are 4 < pH < 6. As we found that pH < 4 is required for the acid-catalyzed reaction, all reaction mixtures with DESs as solvents were chosen that have pH values pH < 4, namely 0.8 < pH < 2.9. This was also the reason why we decided not to investigate standard DES: Aqueous solutions of DESs prepared from ChCl:glycerol (1:2) and ChCl:ethylene glycol (1:2) have rather high pH values of approximately between 4.40 and 4.00, and 4.36 and 4.08, and thus they are not suitable for the fructose dehydration. Given that pH < 4, the solvent decides on the yield, and the pH does not influence the yield any more. Similar observations were found which, however, used extremely acid conditions by toluene sulfonic acid (pKa = −2.8) (Assanosi et al., 2014). Thus, given that pH is low enough (pH < 3 is recommended) and the reaction is catalyzed meaningfully, the kind of the solvent determines the equilibrium yield of HMF. In order to validate this hypothesis, the influence of acidity on the HMF yield was investigated in this work. For this purpose, the reaction was carried out in a DES consisting of constant HBA while varying the HBD under equal reaction conditions. The salt tetraethyl ammonium chloride (TEAC) served as HBA in this study combined with the HBDs levulinic acid (LA) or lactic acid (LAA). All DESs were prepared with a molar ratio of TEAC:HBD 1:2. The so-obtained HMF yields and selectivity are illustrated in Figure 2.

After 48 h, an HMF yield of 29% was achieved in TEAC:LAA compared to 45% in TEAC:LA. Thus, using levulinic acid as HBD allows faster kinetics. Even more, also the reaction equilibrium (TEAC:LA 68% yield) was found to be favored in levulinic acid based DESs over lactic acid based DESs (TEAC:LAA 57% yield). The results shown in Figure 2 mean that the DES containing the weaker acid (levulinic acid) allows a faster reaction and even a higher yield of HMF. This is unexpected since it disproves the hypothesis that very acidic conditions are required for the considered reaction (Chheda et al., 2007). Rather, the molecular interactions caused by the solvents are the reason for higher yield and faster kinetics of the reaction. This has been found for diverse reactions in the literature (Lemberg and Sadowski, 2017; Voges et al., 2017; Wangler et al., 2019).

The finding that acidity plays a minor role is further studied by analyzing the relation between pH and HMF yield. pH values in various DESs was measured with the pH meter Qph 70 (VWR International GmbH, Langenfeld, Germany). The measurements were carried out three times at 295 K. These averaged pH values are listed in Table 3.

**Table 3:** The pH values of the different DESs at 295 K and 1 bar.

**DES**	**ChCl:LA**	**TBAC:LA**	**TEAC:LA**	**ChCl:LAA**	**TBAC:LAA**	**TEAC:LAA**
pH [–]	1.62 ± 0.04	1.71 ± 0.04	2.92 ± 0.06	0.48 ± 0.01	0.83 ± 0.02	1.37 ± 0.01

*Standard uncertainty: u(T) = 0.5 K; u(pH) = 0.02*.

**Figure 3:**
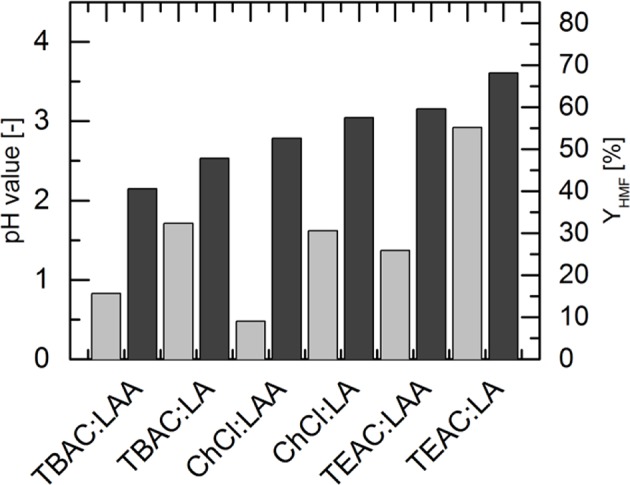
The HMF yields (black bars) as well as the pH-values (gray bars) in the different deep eutectic solvents with molar ratio HBA:HBD 1:2. The HMF yields were achieved in the DESs consisting of the quaternary ammonium salts and levulinic acid or lactic acid. Data listed in Table S4.

In both the LA-based DESs and in the LAA-based DESs, the pH can be sequenced in the same order of the HBA quaternary ammonium salts: pH(TEAC-based DES) > pH(TBAC-based DES) > pH(ChCl-based DES). Due to the carboxylic acids used, the pH in the DESs with LAA as HBD is comparatively lower than in the DESs with LA as HBD. The HMF yield and the pH obtained in the DESs are plotted in Figure 3.

It can be observed from Figure 3 that pH-value of the DESs does not correlate with the achieved HMF yield. This result was obtained for both LA-based DESs and LAA-based DESs. Thus, it can be concluded that the chemical nature of the HBA itself leads to the different reactivities in the reaction under investigation. This is further investigated in the next set of experiments.

### Influence of the Chemical Nature of the HBA in DESs With Levulinic Acid

**Figure 4:**
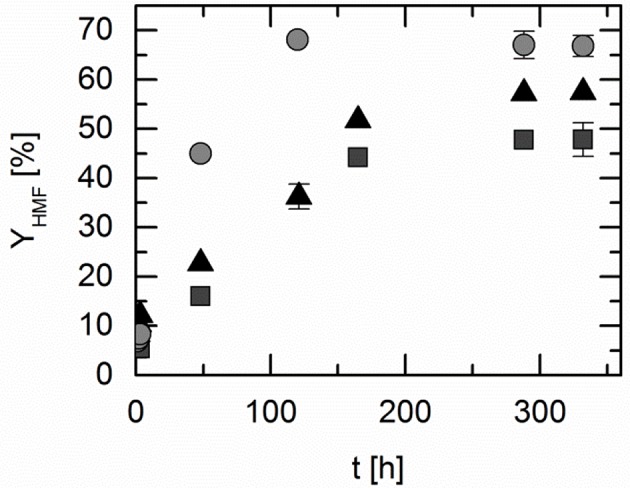
5-Hydroxymethylfurfural (HMF) yield over time in the different deep eutectic systems based on levulinic acid (LA); reaction temperature 323 K; w_FRU,0_ = 2.5 wt.%; pressure 1 bar. Symbols are yields in different DESs: triangles: choline chloride (ChCl):LA 1:2; squares: tetrabutyl ammonium chloride (TBAC):LA 1:2; circles: tetraethyl ammonium chloride (TEAC):LA 1:2. Data listed in Table S5.

The experiments in LA-based DESs were performed as described in the section Experiments and Methods. The results of the two independent samples were measured in triplicate and are shown in Figure 4, which presents the influence of the HBA constituent on the HMF yield. Three different HBAs were investigated: choline chloride (ChCl), tetrabutyl ammonium chloride (TBAC), and tetraethyl ammonium chloride (TEAC).

In ChCl:LA the HMF yield after 3 h reached 12%, in TBAC:LA 5% and in TEAC:LA 8%. After 120 h, maximum yield (equilibrium) was reached in TEAC: LA of 68%. In both, ChCl:LA and TBAC:LA equilibrium was reached after 312 h with maximum yields of HMF of 57% (ChCl:LA) and of 48% (TBAC:LA). The maximum yield of HMF increased from TBAC as HBA, through ChCl to TEAC, as it was already observed in the LAA-based DESs (Table S6). These results indicate a significant influence of hydrophilicity on HMF yield. This again points out the finding that molecular interactions induced by the solvent are responsible for the findings in this work.

### Reactivity of Reaction Medium Containing DES and Additional Vanadium Catalyst

**Figure 5:**
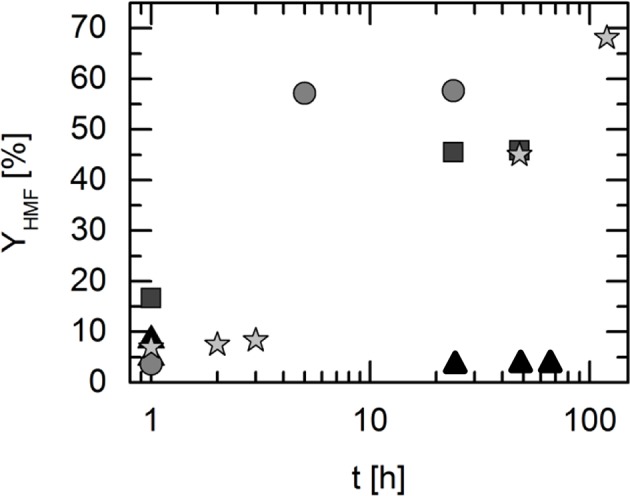
5-Hydroxymethylfurfural (HMF) yield over time in the deep eutectic systems consisting of tetraethyl ammonium chloride and levulinic acid (TEAC:LA 1:2) with different catalysts. The catalysts were added to the fructose+DES mixture undissolved as a powder. Reaction temperature: 323 K; w_FRU,0_ = 2.5 wt.%. Symbols present yields in different reaction medium: triangles: TEAC:LA with NH_4_VO_3_; squares: TEAC:LA with VOSO_4_; circles: TEAC:LA with HPA-5; stars: TEAC:LA without catalyst. Data listed in Table S7.

Due to the large time span required to reach the reaction equilibrium in the investigated systems, the use of different vanadium catalysts was further investigated. The selection of promising homogeneous vanadium-based catalysts was done based on former investigations carried out by Albert et al. (2019). The influence of the catalysts on the reaction kinetics was determined using the DES that allowed highest yields of HMF without additional catalyst. In this work this was found to be the DES TEAC:LA in a molar ratio of 1:2 at a reaction temperature of 323 K. The experiments were carried out as described in section Synthesis of Deep Eutectic Solvents. In the following, a sample was taken every hour at the beginning and analyzed immediately. In each case the same amount of catalyst was added as a powder to the standard reaction mixture consisting of 2.5 wt% fructose in of DES. Figure 5 shows the HMF yields obtained in TEAC:LA with and without additional vanadium catalyst.

The maximum reachable yield for the reaction in the solvent TEAC:LA is illustrated in Figure 5 yielding Y_HMF_ = 68%. This value is the thermodynamically limited equilibrium yield, reached after about 100 h. Using additional catalysts allows improving the kinetics, which is shown clearly in Figure 5. In the solvent TEAC:LA + NH_4_VO_3_, the yield reached 5% (3%) after 1 h (24 h) of reaction time. Using the solvent TEAC:LA + VOSO_4_, the HMF yield was found to be 17% (45%) after 1 h (24 h) of reaction time. After 24 h, the yield did not further increase in this solvent. In comparison to the above-used vanadium salts, the addition of HPA-5 as a catalyst to the solvent TEAC:LA allows an HMF yield of 57% after 5 h. This was the maximally achievable yield at the applied conditions. Thus, the heteropolyacid HPA-5 drastically increased reaction kinetics and might be a very promising perspective for future investigations. Furthermore, high HMF selectivities (see Figure S2) could be reached demonstrating the beneficial effect of the DES:POM reaction system. Nevertheless, still the yield does not increase higher than Y_HMF_ = 57%, even not after 5 h of time. The yield was measured in HPA-5 + TEAC:LA up to 48 h (see Figure S5). The reason behind the difference to the maximum achievable yield (Y_HMF_ = 68%) is probably caused by solvent/catalyst interactions. For samples that contained HPA-5 a color change of the reaction solution was observed turning from yellowish to dark brownish with increasing reaction time (see Figure S4). This might be due to side product (mostly humin) formation as described earlier. Obviously, this limits the maximum achievable HMF yield using this catalyst system without product isolation. This underlines the necessity to isolate the product (and especially water as the side product) from the reaction solution as it further decreases the pH value that favors undesired humin formation.

As a remark, it shall be stated that the total achievable HMF yield at thermodynamic equilibrium is 57% using the DES consisting of ChCL:LA, while the highest yield was found for the DES TEAC:LA. Even though the reaction conditions used in this study are mild, these yields are still moderate and the reaction time without catalyst is very long especially (cf. more than 300 h in Figure 1) to reach the equilibrium yield. In the literature, higher yields were obtained at cost of more toxic conditions and higher energy required (Vandam et al., 1986; Chheda et al., 2007; Hu et al., 2008; Hansen et al., 2009; Ilgen et al., 2009; Thi et al., 2010; Russ and Konig, 2012; D'Anna et al., 2014; Yi et al., 2015; Zhang et al., 2015).

### Solvent Recycling

**Figure 6:**
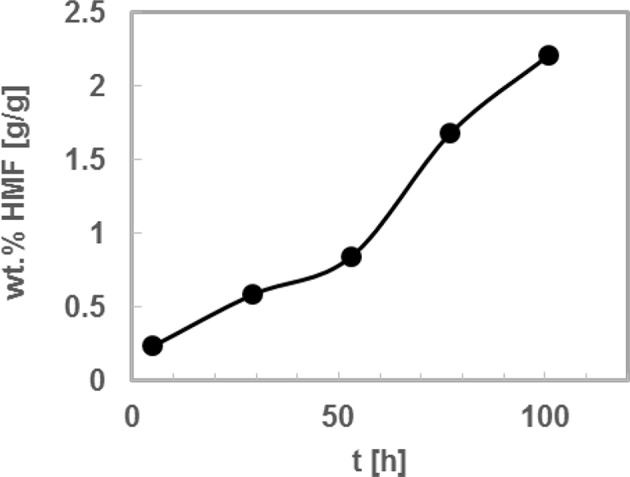
5-Hydroxymethylfurfural (HMF) weight fraction over time in the deep eutectic systems consisting of tetraethyl ammonium chloride and levulinic acid (TEAC:LA 1:2) with HPA-5 catalyst. Four wt% of the catalyst was added at *t* = 0 to the fructose+DES mixture undissolved as a powder. Reaction temperature: 323 K; w_FRU,0_ = 2.5 wt.%. Symbols present data upon addition of 2.5 wt% fructose after each reaction step.

As a final step the solvent recyclability was investigated for the best system, i.e., TEAC-LA (1:2) in combination with 4 wt. % HPA-5 at 323 K. For this step, the initial reaction mixture was prepared once, and after defined times the reactant fructose was added again. It should be mentioned that no new catalyst was added. That is, the DES solvent was used for five subsequent times and after about 5 h of reaction the concentration of HMF was measured prior to adding another 2.5 w% of fructose only. The results are shown in Figure 6. It can be observed that a nearly linear increase of HMF weight fraction was obtained. That is, adding fructose at defined reaction time allows similar conversion of fructose independent of the HMF that was subsequently produced during the single reaction steps. To conclude, the solvent could be successfully used for five times in a row without losing its efficiency at the conditions under investigation. Please note that we did not investigate any additional cycle of fructose addition.

The exact procedure we carried out can be described as “Recycling without product isolation,” which is standard procedure in chemical reaction engineering. We have recently developed methods to extract HMF from aqueous solutions using hydrophobic solvents such as ketones (Mohammad et al., 2016) or hydrophobic DESs (Dietz et al., 2019). Thus, a meaningful extraction method is to feed the product with hydrophobic solvent (ketone or DES), upon which the HMF will be separated into a second phase, and then to distillate the mixture (in case of ketone as extractant the ketone will be the head product). Further, an increase of the fructose concentration is of course an important step for an upscale of the process. However, the limited fructose solubility in the considered DESs might be a limitation toward concentration increase. We measured experimentally the fructose solubilities in the considered DESs. It can be observed that solubility in LAA– based DESs are higher than in LA-based DESs. However, the HMF yields in the LAA– based DESs are lower than in LA-based DESs. The solubilities are presented in Table S9.

## Conclusions

The conversion of biomass to valuable products is of utmost importance to replace petroleum-based chemical syntheses. In this contribution, we studied the reaction from fructose to HMF as a key reaction and as a model reaction in which water is formed as product. From thermodynamic point-of-view, water as reaction medium has to be avoided for high conversion. Nevertheless, the conversion is acid-catalyzed. Thus, we used acidic deep eutectic solvents (DES) as a reaction medium, and due to their low thermal stability we focused on low reaction temperatures of 323 K. The used DESs were chosen to be liquid at this reaction temperature, which we measured and also predicted with thermodynamic models. Further, the used DESs had to behave acidic and thus we studied DESs based on carboxylic acids, in this work the biogenic levulinic acid and lactic acid.

The results showed that the DESs based on the combination of a chloride salt (e.g., choline chloride) and of a carboxylic acid are promising reaction medium for the synthesis of HMF. Maximum yields of about 70% were obtained at 323 K. A detailed study on the influence of the kind of DES revealed that temperature (up to 343 K) and acidity in the reaction mixtures (0.4 < pH < 3.5) as well as the composition of the DES (i.e., the ratio between chloride salt and carboxylic acid) do little influence the maximum achieved yield. In contrast, the kind of the DES constituents strongly influenced the measured yield. The use of DESs composed of levulinic acid instead of lactic acid as well as of tetraethyl ammonium chloride instead of choline chloride or tetrabutyl ammonium chloride allowed higher yields. As the reaction reached high yields after long reaction times, the kinetics were increased in this work using different vanadium-based catalysts. The polyoxometalate HPA-5 (H_8_PV_5_Mo_7_O_40_) lead to drastically increased reaction kinetics giving a maximum HMF yield of about 60% after only 5 h reaction time without decreasing the very high product selectivity. The reaction solvent (DES+HPA-5) could be successfully used for five times in a row without losing its efficiency at the conditions under investigation We consider this to be very important step toward sustainable green processing of biomass in the future.

## Data Availability Statement

All datasets generated for this study are included in the manuscript/Supplementary Files.

## Author Contributions

CH and JA: conceptualization. CH and JA: methodology. SK: validation. All authors: formal analysis. SK: investigation. CH and JA: resources. SK: data curation. SK: writing—original draft preparation. CH and JA: writing—review and editing. SK: visualization. CH: supervision. CH: project administration. CH: funding acquisition.

### Conflict of Interest

The authors declare that the research was conducted in the absence of any commercial or financial relationships that could be construed as a potential conflict of interest.

## References

[B1] Abbott A. P. Boothby D. Capper G. Davies D. L. Rasheed R. K. (2004). Deep eutectic solvents formed between choline chloride and carboxylic acids: versatile alternatives to ionic liquids. J. Am. Chem. Soc. 126, 9142–9147. 10.1021/ja048266j 15264850

[B2] Albert J. Luders D. Bosmann A. Guldi D. M. Wasserscheid P. (2014). Spectroscopic and electrochemical characterization of heteropoly acids for their optimized application in selective biomass oxidation to formic acid. Green Chem. 16, 226–237. 10.1039/C3GC41320A

[B3] Albert J. Mehler J. Tucher J. Kastner K. Streb C. (2016). One-step synthesizable lindqvist-isopolyoxometalates as promising new catalysts for selective conversion of glucose as a model substrate for lignocellulosic biomass to formic acid. Chem. Select 1, 2889–2894. 10.1002/slct.201600797

[B4] Albert J. Mendt M. Mozer M. Voss D. (2019). Explaining the role of vanadium in homogeneous glucose transformation reactions using NMR and EPR spectroscopy. Appl. Catalysis A General 570, 262–270. 10.1016/j.apcata.2018.10.030

[B5] Albert J. Wolfel R. Bosmann A. Wasserscheid P. (2012). Selective oxidation of complex, water-insoluble biomass to formic acid using additives as reaction accelerators. Energy Environ Sci. 5, 7956–7962. 10.1039/c2ee21428h

[B6] Altuntepe E. Emel'yanenko V. N. Forster-Rotgers M. Sadowski G. Verevkin S. P. Held C. (2017). Thermodynamics of enzyme-catalyzed esterifications: II. Levulinic acid esterification with short-chain alcohols. Appl. Microbiol. Biotechnol. 101, 7509–7521. 10.1007/s00253-017-8481-4 28905090

[B7] Ammam M. (2013). Polyoxometalates: formation, structures, principal properties, main deposition methods and application in sensing. J. Mater. Chem. A 1, 6291–6312. 10.1039/c3ta01663c

[B8] Assanosi A. A. Farah M. M. Wood J. Al-Duri B. (2014). A facile acidic choline chloride-p-TSA DES-catalysed dehydration of fructose to 5-hydroxymethylfurfural. RSC Adv. 4, 39359–39364. 10.1039/C4RA07065H

[B9] Bozell J. J. Petersen G. R. (2010). Technology development for the production of biobased products from biorefinery carbohydrates—the US Department of Energy's “Top 10” revisited. Green Chem. 12, 539–554. 10.1039/b922014c

[B10] Carniti P. Gervasini A. Marzo M. (2011). Absence of expected side-reactions in the dehydration reaction of fructose to HMF in water over niobic acid catalyst. Catalysis Commun. 12, 1122–1126. 10.1016/j.catcom.2011.03.025

[B11] Chheda J. N. Román-Leshkov Y. Dumesic J. A. (2007). Production of 5-hydroxymethylfurfural and furfural by dehydration of biomass-derived mono- and poly-saccharides. Green Chem. 9, 342–350. 10.1039/B611568C

[B12] Crespo E. A. Silva L. P. Martins M. A. R. Bulow M. Ferreira O. Sadowski G. (2018). The role of polyfunctionality in the formation of [ch]cl-carboxylic acid-based deep eutectic solvents. Ind. Eng. Chem. Res. 57, 11195–11209. 10.1021/acs.iecr.8b01249

[B13] D'Anna F. Marullo S. Vitale P. Rizzo C. Lo Meo P. Noto R. (2014). Ionic liquid binary mixtures: promising reaction media for carbohydrate conversion into 5-hydroxymethylfurfural. Appl. Catalysis A General 482, 287–293. 10.1016/j.apcata.2014.05.039

[B14] Dietz C. H. J. T. Gallucci F. van Sint Annaland M. Held C. Kroon M. C. (2019). 110th anniversary: distribution coefficients of furfural and 5-hydroxymethylfurfural in hydrophobic deep eutectic solvent + water systems: experiments and perturbed-chain statistical associating fluid theory predictions. Ind. Eng. Chem. Res. 58, 4240–4247. 10.1021/acs.iecr.8b06234

[B15] Francisco M. van den Bruinhorst A. Kroon M. C. (2013). Low-transition-temperature mixtures (lttms): a new generation of designer solvents. Angew. Chem. Int. Ed. 52, 3074–3085. 10.1002/anie.201207548 23401138

[B16] Garcia-Alvarez J. Hevia E. Capriati V. (2018). The future of polar organometallic chemistry written in bio-based solvents and water. Chemistry 24, 14854–14863. 10.1002/chem.201802873 29917274

[B17] Hansen T. S. Woodley J. M. Riisager A. (2009). Efficient microwave-assisted synthesis of 5-hydroxymethylfurfural from concentrated aqueous fructose. Carbohydrate Res. 344, 2568–2572. 10.1016/j.carres.2009.09.036 19850284

[B18] Hu S. Zhang Z. Zhou Y. Han B. Fan H. Li W. (2008). Conversion of fructose to 5-hydroxymethylfurfural using ionic liquids prepared from renewable materials. Green Chem. 10, 1280–1283. 10.1039/b810392e

[B19] Ilgen F. Ott D. Kralisch D. Reil C. Palmberger A. König B. (2009). Conversion of carbohydrates into 5-hydroxymethylfurfural in highly concentrated low melting mixtures. Green Chem. 11, 1948–1954. 10.1039/b917548m

[B20] Istasse T. Bockstal L. Richel A. (2018). Production of 5-hydroxymethylfurfural from D-fructose in low-transition-temperature mixtures enhanced by chloride anions and low amounts of organic acids. ChemPlusChem 83, 1135–1143. 10.1002/cplu.201800416 31950705

[B21] Iwata T. (2015). Biodegradable and bio-based polymers: future prospects of eco-friendly plastics. Angew. Chem. Int. Ed. 54, 3210–3215. 10.1002/anie.201410770 25583677

[B22] Kozhevnikov I. V. (1998). Catalysis by heteropoly acids and multicomponent polyoxometalates in liquid-phase reactions. Chem. Rev. 98, 171–198. 10.1021/cr960400y 11851502

[B23] Kuster B. F. M. van der Baan S. H. (1977). The influence of the initial and catalyst concentrations on the dehydration of d-fructose. Carbohydrate Res. 54, 165–176. 10.1016/S0008-6215(00)84806-5

[B24] Lemberg M. Sadowski G. (2017). Predicting the solvent effect on esterification kinetics. ChemPhysChem 18, 1977–1980. 10.1002/cphc.201700507 28557330

[B25] Li G. B. Jiang Y. T. Liu X. B. Deng D. S. (2016). New levulinic acid-based deep eutectic solvents: synthesis and physicochemical property determination. J. Mol. Liquids 222, 201–207. 10.1016/j.molliq.2016.07.039

[B26] Liu F. Barrault J. Vigier K. D. Jerome F. (2012). Dehydration of highly concentrated solutions of fructose to 5-hydroxymethylfurfural in a cheap and sustainable choline chloride/carbon dioxide system. ChemSusChem 5, 1223–1226. 10.1002/cssc.201200186 22644952

[B27] Mohammad S. Held C. Altuntepe E. Köse T. Sadowski G. (2016). Influence of salts on the partitioning of 5-hydroxymethylfurfural in water/MIBK. J. Phys. Chem. B 120, 3797–3808. 10.1021/acs.jpcb.5b11588 27027570

[B28] Odyakov V. F. Zhizhina E. G. (2008). A novel method of the synthesis of molybdovanadophosphoric heteropoly acid solutions. Reaction Kinetics Catalysis Lett. 95, 21–28. 10.1007/s11144-008-5374-7

[B29] Okano T. Qiao K. Bao Q. X. Tomida D. Hagiwara H. Yokoyama C. (2013). Dehydration of fructose to 5-hydroxymethylfurfural (HMF) in an aqueous acetonitrile biphasic system in the presence of acidic ionic liquids. Appl. Catalysis A General 451, 1–5. 10.1016/j.apcata.2012.11.004

[B30] Russ C. Konig B. (2012). Low melting mixtures in organic synthesis - an alternative to ionic liquids? Green Chem. 14, 2969–2982. 10.1039/c2gc36005e

[B31] Sheldon R. A. (2016). Biocatalysis and biomass conversion in alternative reaction media. Chemistry 22, 12983–12998. 10.1002/chem.201601940 27383560

[B32] Smith E. L. Abbott A. P. Ryder K. S. (2014). Deep eutectic solvents (DESs) and their applications. Chem. Rev. 114, 11060–11082. 10.1021/cr300162p 25300631

[B33] Tang X. Zuo M. Li Z. Liu H. Xiong C. X. Zeng X. H.. (2017). Green processing of lignocellulosic biomass and its derivatives in deep eutectic solvents. ChemSusChem 10, 2696–2706. 10.1002/cssc.201700457 28425225

[B34] Thi P. T. P. Cho C. W. Yun Y. S. (2010). Environmental fate and toxicity of ionic liquids: a review. Water Res. 44, 352–372. 10.1016/j.watres.2009.09.030 19854462

[B35] Vandam H. E. Kieboom A. P. G. Vanbekkum H. (1986). The conversion of fructose and glucose in acidic media—formation of hydroxymethylfurfural. Starch Starke 38, 95–101. 10.1002/star.19860380308

[B36] Vitale P. Abbinante V. M. Perna F. M. Salomone A. Cardellicchio C. Capriati V. (2017). Unveiling the hidden performance of whole cells in the asymmetric bioreduction of aryl-containing ketones in aqueous deep eutectic solvents. Adv. Synthesis Catalysis 359, 1049–1057. 10.1002/adsc.201601064

[B37] Voges M. Fischer C. Wolff D. Held C. (2017). Influence of natural solutes and ionic liquids on-the yield of enzyme-catalyzed reactions: measurements and predictions. Organic Process Res. Dev. 21, 1059–1068. 10.1021/acs.oprd.7b00178

[B38] Wang S. S. Yang G. Y. (2015). Recent advances in polyoxometalate-catalyzed reactions. Chem. Rev. 115, 4893–4962. 10.1021/cr500390v 25965251

[B39] Wangler A. Loll R. Greinert T. Sadowski G. Held C. (2019). Predicting the high concentration co-solvent influence on the reaction equilibria of the ADH-catalyzed reduction of acetophenone. J. Chem. Thermodyn. 128, 275–282. 10.1016/j.jct.2018.08.021

[B40] Werpy T. Petersen G. (2004). Top Value Added Chemicals from Biomass: Volume I—Results of Screening for Potential Candidates from Sugars and Synthesis Gas. Springfield, VA: U.S. Department of Energy 10.2172/15008859

[B41] Yi X. H. Delidovich I. Sun Z. Wang S. T. Wang X. H. Palkovits R. (2015). A heteropoly acid ionic crystal containing Cr as an active catalyst for dehydration of monosaccharides to produce 5-HMF in water. Catalysis Sci. Technol. 5, 2496–2502. 10.1039/C4CY01555J

[B42] Zakrzewska M. E. Bogel-Lukasik E. Bogel-Lukasik R. (2011). Ionic liquid-mediated formation of 5-hydroxymethylfurfural-a promising biomass-derived building block. Chem. Rev. 111, 397–417. 10.1021/cr100171a 20973468

[B43] Zhang J. Yu X. X. Zou F. X. Zhong Y. H. Du N. Huang X. R. (2015). Room-temperature ionic liquid system converting fructose into 5-hydroxymethylfurfural in high efficiency. ACS Sustain. Chem. Eng. 3, 3338–3345. 10.1021/acssuschemeng.5b01015

[B44] Zhang Q. H. Vigier K. D. Royer S. Jerome F. (2012). Deep eutectic solvents: syntheses, properties and applications. Chem. Soc. Rev. 41, 7108–7146. 10.1039/c2cs35178a 22806597

[B45] Zhao B. Y. Xu P. Yang F. X. Wu H. Zong M. H. Lou W. Y. (2015). Biocompatible deep eutectic solvents based on choline chloride: characterization and application to the extraction of rutin from *Sophora japonica*. ACS Sustain. Chem. Eng. 3, 2746–2755. 10.1021/acssuschemeng.5b00619

[B46] Zubeir L. F. Held C. Sadowski G. Kroon M. C. (2016). PC-SAFT modeling of CO_2_ solubilities in deep eutectic solvents. J. Phys. Chem. B 120, 2300–2310. 10.1021/acs.jpcb.5b07888 26814164

