# Supplementary material for: Catalytic Low-Temperature Dehydration of Fructose to 5-Hydroxymethylfurfural Using Acidic Deep Eutectic Solvents and Polyoxometalate Catalysts
Source: Front Chem. 2019 Oct 9;7:661. doi: 10.3389/fchem.2019.00661 (PMC6794411; doi:10.3389/fchem.2019.00661)
Supplement: Supplementary file 1 [file Table_1.docx]

***Supplementary Material***

**
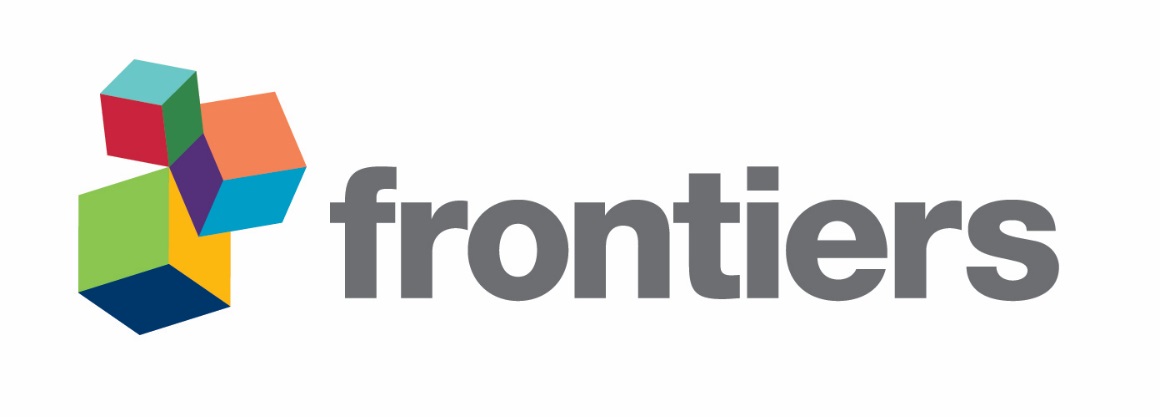
**

Table SI-1: Experimentally determined retention times of all analyzed components, and if they are present as solids at 298 K, the solvents used. All retention times were measured at a flow rate of 0.6 ml/min as well as at 308 K column and 298 K measuring cell temperature.

| Components | Solvent | Retention time [min] |
| --- | --- | --- |
| Water | - | 6.5 |
| Choline chloride (ChCl) | Water | 6.5 |
| Tetraethyl ammonium chloride (TEAC) | Water | 6.6 |
| Tetrabutyl ammonium chloride (TBAC) | Water | 6.7 |
| Fructose | Water | 9.8 |
| Lactic acid (LAA) | - | 13.7 |
| Levulinic acid (LA) | - | 17.4 |
| 5-hydroxymethylfurfural (HMF) | Water | 36.3 |

Standard uncertainity: u(V) = 10 µl/min.

Table SI-2: 5-Hydroxymethylfurfural (HMF) yields in choline chloride:water (ChCl:W), levulinic acid (LA), and choline chloride:levulinic acid (ChCl:LA); reaction temperature: 323 K; fructose starting concentration: 2.5 wt.%; pressure: 1 bar.

| time [h] | ChCl:W | | LA | | ChCl:LA | |
| --- | --- | --- | --- | --- | --- | --- |
|  | Y_HMF_ [%] | SD [%] | Y_HMF_ [%] | SD [%] | Y_HMF_ [%] | SD [%] |
| 1 | - | - | - | - | 8.31 | ^(a)^ |
| 2 | - | - | - | - | 10.31 | ^(a)^ |
| 3 | - | - | - | - | 12.18 | ^(a)^ |
| 48 | 0.00 | 0.00 | 14.14 | 0.00 | 22.70 | 1.03 |
| 121 | - | - | 14.38 | 0.00 | 36.25 | 2.54 |
| 165 | 0.00 | 0.00 | 13.86 | 0.00 | 51.71 | 0.44 |
| 288 | - | - | 11.74 | 4.08 | 57.19 | 0.73 |
| 332 | - | - | - | - | 57.46 | 0.13 |

^(a)^ Due to the short time between sampling and the duration of the HPLC analysis, the samples were analyzed only once. Accordingly, no standard deviation could be calculated.

Standard uncertainty: u(m) = 0.15 mg; u(w_H2O_) = 0.03; u(T) = 0.5 K; u(Y_HMF_) = 1.7 %.

Table SI-3: The different deep eutectic systems (DESs), each composed of a hydrogen bond acceptor (HBA) and one of the two carboxylic acids: lactic acid (LAA) and levulinic acid (LA). The maximum 5-hydroxymethylfurfural (HMF) yields are listed in ascending order from left to right. In addition, the pH measured in the various DESs is documented.

| HBA | TBAC | | ChCl | | TEAC | |
| --- | --- | --- | --- | --- | --- | --- |
| HBD | LAA | LA | LAA | LA | LAA | LA |
| Y_HMF_ [%] | 40.6 | 47.8 | 52.6 | 57.5 | 59.6 | 68.1 |
| pH [-] | 0.83 | 1.71 | 0.48 | 1.62 | 1.37 | 2.92 |

Standard uncertainty: u(m) = 0.15 mg; u(wH2O) = 0.03; u(T) = 0.5 K; u(YHMF) = 1.7 %; u(pH) = 0.02.

Table SI-4: 5-Hydroxymethylfurfural (HMF) yields in deep eutectic systems consisting out of tetraethyl ammonium chloride with levulinic (LA) or lactic acid (LAA) in a molar ratio of 1:2; reaction temperature: 323 K; fructose starting concentration: 2.5 wt.%; pressure: 1 bar.

| time  [h] | TEAC:LA | | TEAC:LAA | |
| --- | --- | --- | --- | --- |
|  | Y_HMF_ [%] | SD [%] | Y_HMF_ [%] | SD [%] |
| 1 | 6.92 | ^(a)^ | - | - |
| 2 | 7.46 | ^(a)^ | - | - |
| 3 | 8.36 | ^(a)^ | - | - |
| 48 | 44.94 | 1.19 | 29.42 | 1.53 |
| 121 | 68.11 | - | 57.04 | 3.68 |
| 288 | 67.04 | 2.76 | - | - |
| 312 | - | - | 59.06 | 5.79 |
| 332 | 66.81 | 2.12 | - | - |
| 350 | - | - | 59.55 | 4.92 |

^(a)^ Due to the short time between sampling and the duration of the HPLC analysis, the samples were analyzed only once. Accordingly, no standard deviation could be calculated.

Standard uncertainty: u(m) = 0.15 mg; u(w_H2O_) = 0.03; u(T) = 0.5 K; u(Y_HMF_) = 1.7 %.

Table SI-5: 5-Hydroxymethylfurfural (HMF) yields in deep eutectic systems based on levulinic acid (LA) in a molar ratio of 1:2; reaction temperature: 323 K; fructose starting concentration: 2.5 wt.%; pressure: 1 bar.

| time  [h] | ChCl:LA | | TBAC:LA | | TEAC:LA | |
| --- | --- | --- | --- | --- | --- | --- |
|  | Y_HMF_ [%] | SD [%] | Y_HMF_ [%] | SD [%] | Y_HMF_ [%] | SD [%] |
| 1 | 8.31 | ^(a)^ | 5.32 | ^(a)^ | 6.92 | ^(a)^ |
| 2 | 10.31 | ^(a)^ | 5.32 | ^(a)^ | 7.46 | ^(a)^ |
| 3 | 12.18 | ^(a)^ | 5.42 | ^(a)^ | 8.36 | ^(a)^ |
| 48 | 22.70 | 1.03 | 15.99 | 0.67 | 44.94 | 1.19 |
| 121 | 36.25 | 2.54 | - | - | 68.11 | - |
| 165 | 51.71 | 0.44 | 44.20 | 0.74 | - | - |
| 288 | 57.19 | 0.73 | 47.76 | 0.84 | 67.04 | 2.76 |
| 332 | 57.46 | 0.13 | 47.80 | 3.42 | 66.81 | 2.12 |

^(a)^ Due to the short time between sampling and the duration of the HPLC analysis, the samples were analyzed only once. Accordingly, no standard deviation could be calculated.

Standard uncertainty: u(m) = 0.15 mg; u(w_H2O_) = 0.03; u(T) = 0.5 K; u(Y_HMF_) = 1.7 %.

Table SI-6: 5-Hydroxymethylfurfural (HMF) yields in deep eutectic systems based on lactic acid (LAA) in a molar ratio of 1:2; reaction temperature: 323 K; fructose starting concentration: 2.5 wt.%; pressure: 1 bar.

| time  [h] | ChCl:LAA | | TBAC:LAA | | TEAC:LAA | |
| --- | --- | --- | --- | --- | --- | --- |
|  | Y_HMF_ [%] | SD [%] | Y_HMF_ [%] | SD [%] | Y_HMF_ [%] | SD [%] |
| 48 | 22.12 | 2.86 | 24.95 | 1.83 | 29.42 | 1.53 |
| 121 | 28.72 | 0.88 | 40.23 | 0.73 | 57.04 | 3.68 |
| 168 | 41.07 | 0.39 | - | - | - | - |
| 312 | 52.61 | 1.73 | 40.65 | 1.18 | 59.06 | 5.79 |
| 350 | 52.07 | 1.71 | 40.54 | 1.45 | 59.55 | 4.92 |

Standard uncertainty: u(m) = 0.15 mg; u(w_H2O_) = 0.03; u(T) = 0.5 K; u(Y_HMF_) = 1.7 %.

Table SI-7: 5-Hydroxymethylfurfural (HMF) yields in the deep eutectic system tetraethyl ammonium chloride:levulinic acid (TEAC: LA) in a molar ratio of 1:2 with various catalysts added directly (in powder form); reaction temperature: 323 K; fructose starting concentration: 2.5 wt.%; catalyst concentration: 4.0 wt.%; pressure: 1 bar.

| time  [h] | TEAC:LA+NH_4_VO_3_ | | TEAC:LA+VOSO_4_ | | TEAC:LA+HPA-5 | |
| --- | --- | --- | --- | --- | --- | --- |
|  | Y_HMF_[%] | SD [%] | Y_HMF_[%] | SD [%] | Y_HMF_[%] | SD [%] |
| 1 | 4.89 | ^(a)^ | 16.66 | ^(a)^ | 3.65 | ^(a)^ |
| 5 | - | - | - | - | 57.16 | ^(a)^ |
| 24 | 3.00 | ^(a)^ | 45.47 | ^(a)^ | 57.67 | ^(a)^ |
| 30 | 2.62 | ^(a)^ | - | - | - | ^-^ |
| 48 | 2.55 | ^(a)^ | 45.82 | ^(a)^ | - | ^-^ |
| 72 | 1.71 | ^(a)^ | - | ^-^ | - | ^-^ |
| 170 | 1.84 | ^(a)^ | - | ^-^ | - | - |

^(a)^ Due to the short time between sampling and the duration of the HPLC analysis, the samples were analyzed only once. Accordingly, no standard deviation could be calculated.

Standard uncertainty: u(m) = 0.15 mg; u(w_H2O_) = 0.03; u(T) = 0.5 K; u(Y_HMF_) = 1.7 %.

Table SI-8: Manufacturer, purity and CAS-number of the used chemicals.

| Name | Manufacturer | Purity [wt.%] | CAS-number |
| --- | --- | --- | --- |
| Acetonitrile | VWR International | 99.9 | 75-05-8 |
| Choline chloride | Sigma Aldrich | 98.0 | 67-48-1 |
| CombiTitrant 5 | Merck KGaA | ^(a)^ | ^(a)^ |
| D-(-)-fructose | Sigma Aldrich | 99+ | 57-48-7 |
| Dimethyl sulfoxide | Merck KGaA | 99.9 | 67-68-5 |
| 5-(Hydroxymethyl)furfural | Molekula | 99.1 | 67-47-0 |
| Levulinic acid | Sigma Aldrich | 98.0 | 123-76-2 |
| Malonic acid | Alfa Aesar | 99+ | 141-82-2 |
| (-)-Menthol | Tokyo Chemical Industry | 99+ | 2216-51-5 |
| Methanol | Merck KGaA | 99.9 | 67-56-1 |
| DL-lactic acid | Sigma Aldrich | ~90^(b)^ | 50-21-5 |
| L-lactic acid (anhydrous) | Alfa Aesar | 98.0 | 79-33-4 |
| Deionized water | Merck KGaA | ^(c)^ | ^(c)^ |
| Oxalic acid | Sigma Aldrich | 98.0 | 144-62-7 |
| Sulfuric acid | VWR  International | 99.9^(d)^ | 7664-93-9 |
| Tetrabutyl ammonium chloride | Carbolution | 98.0 | 1112-67-0 |
| Tetraethyl ammonium chloride | Alfa Aesar | 98.0 | 56-34-8 |
| Thymol | Tokyo Chemical Industry | 99.9 | 89-83-8 |

^(a)^ No information available from Merck KGaA.

^(b)^ According to Sigma Aldrich; according to Karl Fischer titration, however, 86.9%.

^(c)^ A 0.22 μm membrane filter (Millipak Express, Merck KGaA, Darmstadt, Germany) ensures particle- and bacteria-free water.

^(d)^ 0.1 mol / l sulfuric acid.

Table SI-9: Experimental measured solubility of fructose (298 K, 1 bar) in DESs and water content of the DESs.

**DES x_fructose_ x_water_
[mol/mol]^a^ [mol/mol] ^b^**

| ChCl:LA | 0.0257 | | 0.123 | |  |
| --- | --- | --- | --- | --- | --- |
| TBAC:LA | 0.0391 | | 0.127 | |  |
| TEAC:LA | 0.0303 | | 0.093 | |  |
| ChCl:LAA | | 0.0806 | | 0.306 | |
| TBAC:LAA | | 0.0360 | | 0.197 | |
| TEAC:LAA | | 0.0479 | | 0.264 | |

a: mol/(mol DES+fructose) b: mol/(mol DES+water)


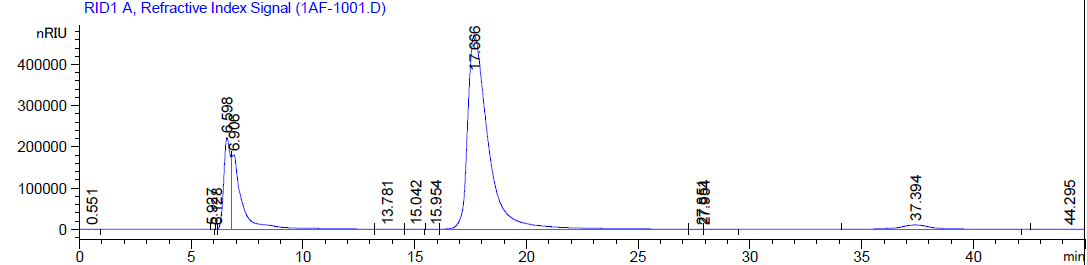


Figure SI-1: HPLC chromatogram (RI – refractive index) of a reaction mixture after a reaction time of 288 hours without additional catalyst. Reaction from fructose to HMF at 323.15 K and 1 bar in the DES TEAC:LA. The peaks belong to tetraethyl ammonium chloride (TEAC; 6.598 min), levulinic acid (LA at 17.666 min) and 5-hydroxymethylfurfural (HMF at 37.394 min). TEAC and water (at 6.906 min) could not be separated by the applied HPLC method. Neither fructose (the substrate; retention time about 9.5 min) nor any other side products were detected.


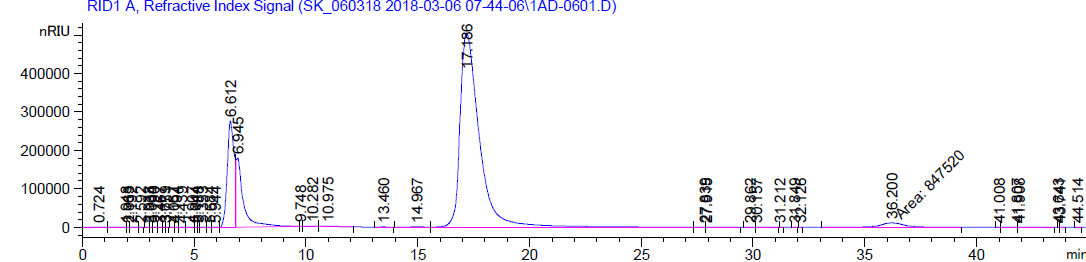


Figure SI-2: HPLC chromatogram (RI – refractive index) of a reaction mixture after a reaction time of 5 hours containing catalyst HPA-5. Reaction from fructose to HMF at 323.15 K and 1 bar in the DES TEAC:LA. The peaks belong to tetraethyl ammonium chloride (TEAC; 6.612 min), levulinic acid (LA at 17.186 min) and 5-hydroxymethylfurfural (HMF at 36.200 min). TEAC and water (at 6.945 min) could not be separated by the applied HPLC method. Neither fructose (the substrate; retention time about 9.5 min) nor any other side products were detected.


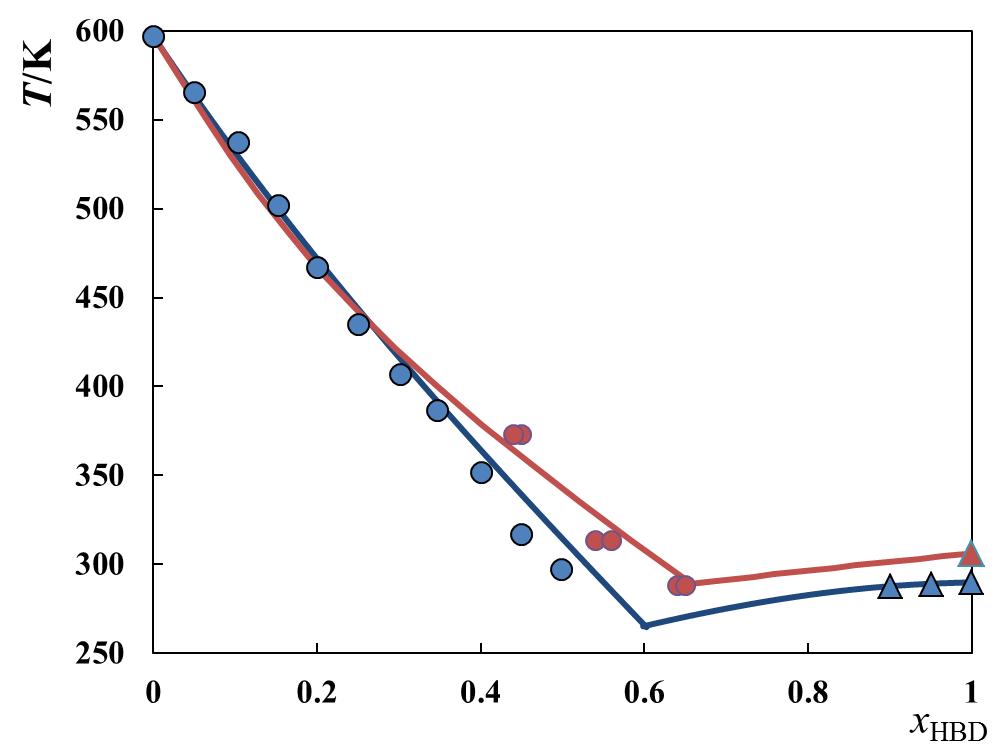


Figure SI-3: Solid-liquid equilibrium between HBD and choline chloride (ChCl) at 1 bar. Red: HBD = levulinic acid (LA), this work. Blue: HBD = DL-lactic acid (LAA), results from literature^[^[^29^](#_ENREF_29)^]^. Symbols are experimental data (circles: ChCl solubility; triangles: HBD solubility), lines are PC-SAFT predictions using the parameters from refs.^[^[^27^](#_ENREF_27)^,^ [^28^](#_ENREF_28)^]^ without using any binary interaction parameters. Solubility modeling required fusion data: enthalpy of fusion (4300 J/mol for ChCl; 11340 J/mol for LAA; 17136 J/mol for LA) and melting temperature (597 K for ChCl; 306 K for LA; 289 K for LAA); heat capacity differences between solid and liquid phase were set to zero for solubility modeling.

Figure SI-4: Coloring of samples of reaction mixtures at 323 K after 1 hour (left sample) and after 48 hours (right sample). The starting weight fraction of fructose was 2.5 w% in the DES tetraethyl ammonium chloride:levulinic acid (TEAC:LA 1:2). Catalyst HPA-5 was added to the reaction mixture undissolved as a powder. The brownish color denotes to the formation of side products, i.e. especially humins.


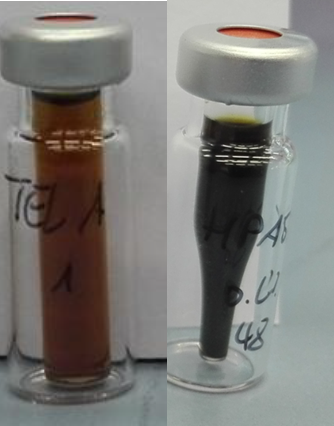


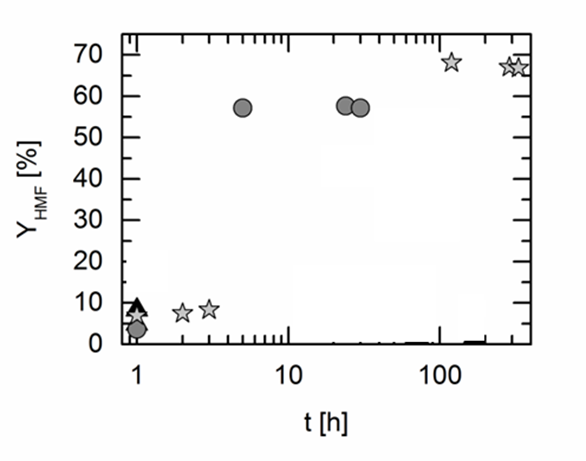


Figure SI-5: 5-Hydroxymethylfurfural (HMF) yield over time in the deep eutectic systems consisting of tetraethyl ammonium chloride and levulinic acid (TEAC:LA 1:2) with different catalysts. The catalysts were added to the fructose+DES mixture undissolved as a powder. Reaction temperature: 323 K; wFRU,0 = 2.5 wt.%. Symbols present yields in different reaction medium: circles: TEAC:LA with HPA 5; stars: TEAC:LA without catalyst.
